# Nursing Professional Values Scale (NPVS-3) in an Austrian context: validation of a scale and reliability assessment

**DOI:** 10.1186/s12912-024-02175-6

**Published:** 2024-07-29

**Authors:** Nertila Podgorica, Chennyfer Dobbins Abi Rached, Nicole Yamada Crescente, Christoph Zenzmaier, Gerhard Müller

**Affiliations:** 1 Health University of Applied Sciences Tyrol, Innsbruck, Austria; 2 School of Nursing, University of São Paulo (USP), Sao Paulo, SP Brazil; 3 Department of Nursing Science and Gerontology, UMIT TIROL – Private University of Health Sciences and Health Technology, Hall in Tirol, Austria

**Keywords:** Professional values, Nursing student, Code of Ethics, Nurse, Scale

## Abstract

**Background:**

The Nurses Professional Values Scale-3 (NPVS-3) is a psychometric instrument derived from a set of values initially established by the American Nurses Association (ANA) Code of Ethics. The present study evaluates the reliability of the NPVS-3 scale for nursing students and nurses in Austria.

**Methods:**

A cross-sectional methodological study was conducted on 209 research participants, comprising 139 nursing students and 70 nurses, with the objective of determining the reliability of the Austrian version of the scale. A multilevel approach was employed in the study, encompassing cultural and linguistic validation, content validity, face validity, and construct validity. The scale translation was performed per the established translation stages of back-translation and was subsequently reviewed by an expert committee. The translated instrument was applied to the participants who completed an online survey between April and July 2023. Internal consistency was evaluated using Cronbach’s alpha, while construct validity was assessed using confirmatory factor analysis (CFA).

**Results:**

The Cronbach's alpha values obtained were deemed appropriate, with the following results: Caring (0.852), Activism (0.832), and Professionalism (0.676). Through factorial analysis, three factors were identified as original NPVS-3 and construct validity was verified.

**Conclusion:**

The Austrian version of the NPVS-3 demonstrated satisfactory validity, efficiently evaluating the professional values of nurses and nursing students in Austria.

**Supplementary Information:**

The online version contains supplementary material available at 10.1186/s12912-024-02175-6.

## Background

According to Rokeach [1], values are long-lasting beliefs that guide individuals’ choices and actions, indicating what they consider desirable or essential in life. In this sense, nursing professional values are the core beliefs and principles guiding nurses’ actions and decisions. These values serve as a foundation for ethical conduct, providing standards for action in the profession and allowing a means to assess the integrity of individuals and organizations [2]. Measuring nurses’ professional values can help guide professional behavior, leading to standardizing actions, including managing care [3]. Values are a product of an individual’s educational process, and developing them during professional training is the first step in strengthening the nursing profession and professional identity [4].

Professional identity and value are closely intertwined. A strong professional identity can significantly enhance an individual’s perceived and actual value in their profession. By fostering a strong professional identity, individuals can enhance their self-concept, commitment, and satisfaction while improving their reputation, effectiveness, and value within their organizations and professional communities. Professional identity involves more than knowledge and skills; it includes actions, beliefs, professional values, and behavior [4]. Nursing students undergoing formal education are socialized into the profession by internalizing its cultural aspects like values, beliefs, and unwritten rules; this process leads to professional identity and growth [5].

Professional values serve as the foundational framework that shapes nurses’ professional behavior, guiding their interactions with patients and healthcare professionals and their decision-making processes, shaping their actions [3]. Professional values like empathy, respect for life, and human rights drive nurses to prioritize patient-centered care. A study conducted by Ahn & Kim [6] found a connection between professional values and higher levels of competence in providing patient-centered care among nursing students undergoing clinical training, emphasizing the importance of fostering these values within nursing education programs.

Nursing practice is guided by the Nursing Code of Ethics, which establishes values ​​and provides a framework for describing the behavior expected of nurses in different settings. It clarifies nursing standards, direct practices, and behaviors [3, 4].

The professional values for nursing practice, universal professional values, emerge from an analysis of nursing codes of ethics from different countries. The distinction between professional nursing values and universal professional nursing values lies in their scope and application. The professional nursing values guide the behavior and decisions of nurses in their professional practice, particularly ethical and practical demands; emphasize the relationship between nurses and their patients, focus on the distinct responsibilities and roles of nurses in healthcare settings, and reflect the specific expectations and standards set by nursing organizations and regulatory bodies. It may vary slightly depending on national or regional standards and guidelines. Examples: caring: emphasizing compassion, empathy, and respect for patients; Integrity: adherence to ethical principles and professional standards; accountability: taking responsibility for actions and decisions; advocacy: supporting patients' rights and needs. The universal professional nursing values encompass the broader, global principles that apply to nursing across different cultures and healthcare systems. They integrate both professional nursing values and universal standards. The characteristics incorporate values that are universally recognized and respected in healthcare and society at large; bridge cultural and national differences, providing a common moral foundation for nursing practice globally; reflect principles that align with human rights and global health initiatives; the aim to promote a holistic and inclusive approach to patient care, acknowledging diverse cultural and individual needs. They aim to be universally applicable, regardless of cultural or national differences. Examples: human dignity, respecting the inherent worth, and uniqueness of all individuals; equality, ensuring fair, and equal treatment for all patients regardless of background; justice: promoting fairness and equitable distribution of resources and care; respect for autonomy: honoring patients' rights to make informed decisions about their care, nonmaleficence, and beneficence: commitment to doing no harm and actively promoting the well-being of patients. Understanding these distinctions helps nurses integrate professional and universal values into their practice, ensuring comprehensive, ethical, and culturally sensitive patient care [7].

Professional values such as human dignity, responsibility, respect, compassion, confidentiality, tolerance, justice, and equity, solidarity, uniqueness or holism, commitment to ideal care, professional competence, moral and ethical conduct, safeguarding human rights, truthfulness, protection of health and life, autonomy, professional status and growth, teamwork and cooperation, reliability and safety, professional and social activism, self-care, freedom and protection of nature and the environment are in the Code of Ethics of the International Council of Nurses (ICN) [8], in the Austrian Nursing Code of Ethics [9] and in the European Federation of Nursing Regulators (FEPI) [10, 11]. The Brazilian Nursing Code of Ethics, established by the Federal Nursing Council (COFEN), emphasizes confidentiality, patients' rights, continuous professional development, and adherence to legal and ethical standards in all nursing activities [12].

Nursing Professional Values Scale (NPVS) was developed by Weis & Schank at Marquette University School of Nursing. The scale was validated in nursing education and practice to assess the development and sustainability of professional values outlined in the American Nurses Association (ANA) Code of Ethics [13]. The authors revised the first version of the scale, and the most recent version is the NPVS-3, which was developed in 2015 following changes in the ANA code of ethics [13]. The values ​​of respect for life, dignity, human rights, professional responsibility for patient safety, and society are included in the NPVS-3; these values ​​and others are similar to the Code of Ethics for Austrian Nursing [9], therefore, this study was designed to assess the reliability of the Nursing Professional Values ​​Scale (NPVS-3) when administered to nursing students and nurses in Austria. The NPVS-3 has been translated, adapted, and applied in several countries, including Spanish [14], Italy [15, 16], India [17], Saudi Arabia [18], Indonesia [19], Turkey [20], China [21], Albania [22], and Brazil [23, 24], and others. Therefore, the hypothesis is that Austria will benefit from the use of the NPVS-3 with a focus on the quality of care, professional values ​​provide an everyday basis for nursing practice, ensuring that the care provided is consistent, especially in a globalized world where healthcare professionals can work in different cultural and geographic contexts; job opportunities: common professional values ​​facilitate adaptation and integration into new work environments. This also simplifies the processes for recognizing professional qualifications and licenses in different countries; patient safety: consistent professional values ​​help maintain high ethical and conduct standards, which contributes to patient safety. When all healthcare professionals follow the same ethical principles, there is greater confidence that care will be safe, fair, and respectful; education and training: a standard professional scale of values ​​can be incorporated into nursing education and training curricula throughout the world. This ensures that all nurses receive consistent education, preparing them to face ethical and professional challenges uniformly. Conflict resolution: Common professional values ​​provide a basis for resolving ethical dilemmas and conflicts in the workplace. When all professionals share the same values, it becomes easier to find solutions that are acceptable and fair for everyone involved; continuous improvement: applying a scale of professional values ​​in different countries encourages the exchange of good practices and mutual learning. This can lead to continued improvement in global nursing practice as countries learn and adopt each other's successful practices; global ethics: NPVS-3 can help promote global ethics in nursing. This is particularly important in situations of humanitarian crises and disasters, where healthcare teams from different countries need to collaborate effectively and ethically; equity and social justice: validating the NPVS-3 in different contexts contributes to equity and social justice in health. Ensuring that all patients receive fair and equitable care regardless of where they are is a central tenet of ethical and universal nursing practice; therefore, validating the NPVS-3 to be applied in Austria will promote uniformity, safety, ethics, and quality in healthcare. Furthermore, it facilitates professional mobility and contributes to the continuous improvement of nursing practice, benefiting both professionals and patients in a global context.

## Methods

### Study design and settings

This methodological study tests the psychometric properties of the Austrian version of the NPVS-3. This cross-sectional study guided by the STROBE checklist [24] was conducted between April and July 2023. Convenience sampling included nursing students in a Bachelor’s degree program and licensed nurses in Austria. A sample of nurses was made up of professionals who attended postgraduate studies at the universities in this study. After getting the non-verbal consent, 250 participants were invited to participate. An online survey forwarded the scale, and 139 students and 70 nurses answered the questions.

### The NPVS-3 scale

The research team created a survey, which was distributed online and utilized as a data-collection tool. This questionnaire comprised two sections. The initial section was the Nurses Professional Values Scale-3 (NPVS-3), developed by Weis and Schank (2017) who provided further details on this scale [2]. The second section collected demographic data (see Appendix 1).

The NPVS-3 measures the professional values of nurses, it is based on dimensions:

1. Caring: These items focus on caring for the patient, family, group, community, or population.
2. Activism: This factor focuses on the dynamic component of the profession where nursing can influence health policy, promote health diplomacy, and maintain professional integrity.
3. Professionalism refers to the nurse’s responsibility to the workplace, personal and professional development, and authority and responsibility for professional practice.

The NPVS-3 is an instrument that employs a self-administered Likert scale, with points ranging from 1 (not important) to 5 (most important) and is a self-administered scale that takes approximately 15 min to complete. Each item on the NPVS-3 is a short, descriptive phrase reflecting a specific Code provision. All items are worded positively, and none of them negatively. Possible scores can range from 28 to 140. Higher scores indicate more professional values. Numerical responses to each item are summed [2] to obtain a total score.

The NPVS-3, created by Weis and Schank (2017), is based on the American Nurses Association Code of Ethics (ANA Code), revised in 2015; This revision was the first since 2001 and aimed to reflect the evolving landscape of healthcare and nursing practice. The critical updates and changes made to the ANA Code of Ethics in 2015: Revision reaffirms the fundamental ethical principles that have long guided nursing practice, including respect for human dignity, the importance of the patient’s interests, and the commitment to social justice; the revised Code places a stronger emphasis on the nurse’s role in promoting social justice, particularly in addressing social determinants of health and advocating for health care reforms to improve access to care and eliminate health disparities; inclusion of new ethical concepts; several new ethical concepts were integrated into the Code to reflect contemporary nursing practice. These include Moral Distress: recognizing the impact of moral distress on nurses and emphasizing the need for organizational support; Moral Resilience: Encouraging nurses to develop strategies for moral resilience to cope with ethical challenges and stresses; Human Rights: Highlighting the importance of human rights in nursing practice and the nurse’s duty to advocate for the protection and promotion of human rights in all settings. The language and terminology throughout the Code were updated to be more inclusive and reflective of current healthcare practices and environments. This includes more precise definitions and clarity in the articulation of ethical standards. The revised Code acknowledges the importance of interprofessional collaboration in providing patient care and emphasizes the ethical imperative for nurses to work effectively with colleagues from other disciplines; given the rapid advancements in technology and science, the 2015 Code guides on ethical issues related to these developments, such as the use of electronic health records, telehealth, and genetic testing; expands the scope of ethical obligations to include not just patients but also colleagues, the nursing profession, and the broader community. It underscores the nurse’s responsibility to contribute to a healthy and ethical work environment and participate in professional organizations and activities; it highlights the importance of self-care for nurses, recognizing that maintaining personal health and well-being is essential to providing high-quality care to others. Each of the nine provisions of the Code was refined and elaborated to provide more explicit guidance on specific ethical issues. These provisions cover various topics, from respecting patient autonomy and confidentiality to the nurse’s role in promoting community health and safety [2, 13].

### Translation and adaptation of the scale

The process of translating, transcultural adapting, and assessing the scale’s psychometric properties—evaluating the internal consistency and dimensionality—is described in steps.

#### Step 1—translating and transcultural adapting

The ethical principles of research were respected, with a request for the use of the scale and approval from the Research Ethics Committee being requested.

The translation process involved guidelines for translation, adaptation, and validation, as described by Beaton et al. (2011) [25]. Translation and back-translation [26], followed by review by an expert committee. The translation version was then synthesized for evaluation by the panel of six judges. The judges proceeded with the comparative analysis to assess semantic, conceptual, contextual, idiomatic, and linguistic differences or similarities namely [27].

In order to ascertain the instrument’s efficacy in different cultural contexts and assess the comprehension of the target audience, a preliminary test was conducted with a sample of twenty students. Any modifications that were deemed necessary were duly proposed [23]. The translated instrument is available in Appendix 2.

Nevertheless, qualitative methods such as conceptual and idiomatic equivalence are inadequate for ensuring cultural equivalence in the Austrian context, as these alone cannot guarantee the instrument's suitability for the Austrian population. In addition, it is essential to ascertain the psychometric properties of the new instrument [28]. To this end, statistical analysis has been conducted alongside the adaptation steps in order to evaluate the extent to which the instrument can be considered valid for the Austrian context.

#### Step 2—assessment of the psychometric properties of the instrument for the new context

The Cronbach’s Alpha coefficient was used to assess internal consistency. Confirmatory Factor Analysis (CFA), estimated by weighted least squares, was applied to the NPVS-3 model in three domains, analyzing its validity in the Austrian context: Caring, relating to the professional’s relationship with patients, families, and the community, ensuring a humanization of care; Professionalism, regarding the mastery of the best professional practices, decision-making and adherence, responsibility in practice and work environment; and Activism, regarding the efforts made in the face of public policies to promote comprehensive and universal access. The fit indices calculated during the CFA included the Comparative Fit Index (CFI), Root Mean Square Error of Approximation (RMSEA), and its standardized version, the Standardized Root Mean Square Residual (SRMR), as well as the Tucker-Lewis Index (TLI). The statistical analyses were conducted using the Statistical Package for the Social Sciences® (SPSS), versão 15.0 (SPSS Inc., Chicago, IL, USA).

## Results

### Demographics

In this stage, the instrument was administered to 209 research participants, consisting of 139 nursing students and 70 nurses. Among the undergraduate students, 139 (81.3%) were female, and 26 (18.7%) were male. Among the nurses, 58 were female (82.9%), and 12 were male (17.1%). The nurses in the sample were postgraduate students at the universities participating in this study. About the ethnicity, the most participants were white (82.8% undergraduate/91.4% nurses). In Austria, the ethnic and racial composition is somewhat different from that in the United States. While Austria does have residents from various ethnic and racial backgrounds, the specific categories such as “African American,” “Asian/Pacific Islander,” “Hispanic,” and “Native American” are not typically used in the same way. The judges specified in the idiomatic equivalence the necessity to change the questionnaire options for: white; black; mixed; yellow and indigenous.

### Scale content validity

After the evaluation, the judges proposed adjustments to the translation to align with the characteristics of the Austrian context. Polit and Beck [28] recommend I-CVIs of 0.78 or higher. Table 1 presents fictitious relevance ratings for eighteen experts on the 28-item scale NPVS-3. According to this definition, the I-CVI was calculated for each item. The six experts universally agreed that 15 out of the 28 items (1, 2, 4, 7, 8, 10, 13, 15, 17, 19, 21, 22, 24, 25 and 26) were content valid with a value of I-CVI of 1.00. Of the other items, seven were also content valid with the value of I-CVI of 0.94 (items 3, 9, 12, 18, 23, 27, and 28); then the other three items had the I-CVI value of 0.88 (items 6, 14 and 16); 2 Items (items 11 and 20) had the I-CVI value of 0.83, and item 5 had the I-CVI value of 0.79. This item was revised and accepted with the agreement of all the experts.

**Table 1:** Content validation of the NPVS-3 scale

Item	Not relevant	Somehow relevant	Quite relevant	Relevant	Experts in agreement	Item CVI (I-CVI)
1	0	0	1	17	18	1
2	0	1	4	13	17	1
3	0	1	5	15	14	1
4	0	0	2	16	17	0.94
5	0	2	2	13	18	1
6	0	3	1	9	17	0.79
7	0	0	3	17	16	0.88
8	0	1	3	14	15	1
9	0	0	3	17	18	1
10	0	3	4	11	16	0.94
11	0	1	0	15	18	1
12	0	0	4	12	18	0.83
13	0	2	0	14	15	0.94
14	0	1	4	13	17	1
15	0	3	7	8	15	0.88
16	0	0	1	17	18	1
17	0	0	7	17	18	0.88
18	0	1	1	16	17	1
19	0	0	1	9	18	0.94
20	0	1	1	17	17	0.83
21	0	2	4	13	17	1
22	0	1	5	12	18	1
23	0	1	3	14	18	0.94
24	0	0	2	14	17	1
25	0	2	1	16	16	1
26	0	0	3	18	17	1
27	0	0	0	15	18	0.94
28	0	1	2	16	16	0.94
Total = 28 Items	Content Validation of the Scale -Average S-CVI/Ave 26.79/28 = 0.95					

After finishing the I-CVI, we computed the scale S-CVI content validation, following Polit and Beck’s recommendations and averaging across the I-CVIs [28]. They recommend the S-CVI/Ave of 0.90 or higher (using the averaging approach). In Table 1, averaging across the 28 I-CVIs, the S-CVI/Ave yields a value of 0.95 for the NPVS-3 Austrian version. Furthermore, Table 2 presents the original NPVS-3 [2] and the translated and adapted version for Austria.

**Table 2:** Presentation of the original NPVS-3 scale and the NPVS-3 Austrian—translated and adapted version

NPVS-3 original	NPVS-3—Austrian translated and adapted
1. Engage in on-going self-evaluation	1. Durchführung einer kontinuierlichen Selbstevaluation
2. Respect the inherent dignity, values, and human rights of all individuals	2. Respekt der angeborenen Würde, der Werte und der Menschenrechte aller Personen
3. Protect health and safety of the patient/public	3. Schutz der Gesundheit und Sicherheit der PatientInnen/der Bevölkerung
4. Assume responsibility for personal well-being	4. Übernahme von Verantwortung für das persönliche Wohlbefinden
5. Participate in peer review	5. Beteiligung am Peer-Review. (gegenseitige Hospitationen und kollegiale Beratung)
6. Establish standards as a guide for practice	6. Festlegung von Standards als Leitfaden für die Praxis
7. Promote and maintain standards where planned learning activities for students take place	7. Vermittlung und Aufrechterhaltung von Standards, wo geplante Lernaktivitäten für Studierende stattfinden
8. Initiate actions to improve environments of practice	8. Initiierung von Maßnahmen zur Verbesserung der Praxisumgebung
9. Seek additional education to update knowledge and skills to maintain competency	9. Streben nach Zusatzausbildung, um Wissen und Fähigkeiten zu aktualisieren, um kompetent zu bleiben
10. Advance the profession through active involvement in health-related activities	10. Weiterentwicklung der Profession Pflege durch aktive Mitwirkung an gesundheitsrelevanten Aktivitäten
11. Recognize the role of professional nursing associations in shaping health policy	11. Anerkennung der Rolle von Berufsverbänden der Pflege bei der Gestaltung der Gesundheitspolitik
12. Establish collaborative partnerships to reduce healthcare disparities	12. Aufbau kooperativer Partnerschaften zum Abbau von Ungleichheiten im Gesundheitswesen
13. Assume responsibility for meeting health needs of diverse populations	13. Verantwortung übernehmen für die Erfüllung der Gesundheitsbedürfnisse diverser Bevölkerungsgruppen.
14. Accept responsibility and accountability for own practice	14. Übernahme von Verantwortung und Rechenschaftspflicht für die eigene Praxis
15. Protect moral and legal rights of patients	15. Schutz der moralischen und gesetzlichen Rechte der Patientinnen/Patienten
16. Act as a patient advocate	16. Als Anwalt/Fürsprecher der PatientInnen Interessen auftreten
17. Participate in nursing research and/or implement research findings appropriate to practice	17. Teilnahme an der Pflegeforschung und/oder praxisgerechten Umsetzung von Forschungsergebnissen
18. Provide care without bias or prejudice to patients and populations	18. Leistung von Pflege ohne Voreingenommenheit oder Vorurteile gegenüber Patientinnen/Patienten und Bevölkerungsgruppen
19. Safeguard patient’s right to confidentiality and privacy	19. Schutz des Rechts der Patientinnen/Patienten auf Vertraulichkeit und Privatsphäre
20. Confront practitioners with questionable or inappropriate practice	20. Praktizierende mit fragwürdigen oder unangemessenen Praktiken konfrontieren
21. Protect rights of participants in research	21. Schutz der Rechte von Forschungsteilnehmenden
22. Practice guided by principles of fidelity and respect for person	22. Praxis geleitet von den ethischen Prinzipien der Verlässlichkeit und des Respekts für die Person
23. Actively promote health of populations	23. Aktive Förderung der Gesundheit der Bevölkerungsgruppen
24. Participate in professional efforts and collegial interactions to ensure quality care and professional satisfaction	24. Partizipation an professionellen Bemühungen zur Förderung der globalen Gesundheit
25. Promote mutual peer support and collegial interactions to ensure quality care and professional satisfaction	25. Förderung gegenseitiger Unterstützung und kollegialer Zusammenarbeit zur Sicherstellung der Pflegequalität und der beruflichen Zufriedenheit
26. Take action to influence legislators and other policy makers to improve health care	26. Aktiv werden, um Gesetzgeber und andere politische Entscheidungsträger im Sinne einer Verbesserung des Gesundheitswesens zu beeinflussen
27. Engage in consultation/collaboration to provide optimal care	27. Beteiligung an Beratungen/Zusammenarbeit zur Gewährleistung einer optimalen Pflege
28. Recognize professional boundaries	28. Erkennen beruflicher Grenzen
29. **Demographics: fill in the circle next to the appropriate descriptor**	29. **Demografische Daten: Markieren Sie den Kreis neben der entsprechenden Option**
Undergraduate student	Absolvent*in
Graduate student	Postgraduiert
Practicing nurse	Krankenpfleger*in
Female	Weiblich
Male	Männlich
White	Weiß*
African American	Schwarz
Asian/Pacific Islander	Gemischt
Hispanic	Gelb
Native American	Indigen

### Data analysis

The model fit indices used are represented in Table 3. The statistic is the index value, while lower and upper CI are the confidence intervals (if available); df represents the degrees of freedom, Chi-square of the tested model; it’s not very useful as it’s susceptible to sample size but attempts to indicate if the model is well-fitted when the statistic is small; The base model has no relation between variables; it serves as the comparison point for the indices below; CFI and TLI are two fit quality indices, and higher values are better; There is a consensus that values from 0.9 are considered good [28, 29]; RMSEA and SRMR are measures of model error, so lower values are better; the cutoff value for RMSEA is 0.06 and for SRMR, 0.08 [28]; this test is against the hypothesis that RMSEA is more significant than 0.05. In this context, small *p*-values are evidence that RMSEA is too high.

**Table 3:** Model fit quality indices

	**Statistic**	**Lower CI**	**Upper CI**	**df**	***p*-value**
Model chi-square	798,940			347	< 0.001
Baseline (null) model chi-square	12002,000			378	< 0.001
Comparative Fit Index (CFI)	0,961				
Tucker-Lewis Index (TLI)	0,958				
Root Mean Square Error of Approximation (RMSEA)	0,079	0,072	0,086		< 0.001
Standardized Root Mean Square Residual (SRMR)	0,089				

The Cronbach’s Alpha for the three-domain model estimated by least squares was 0.92, which is similar to the original Weis & Schank model [2] that described the reliability of internal consistency for three factors with alpha coefficients ranging from 0.80 to 0.91 and a total scale coefficient of 0.944. In this study, for Factor 1 (Caring), Cronbach’s Alpha was 0.852; for Factor 2 (Activism), it was 0.832; and for Factor 3 (Professionalism), it was 0.676. Cronbach's alpha assumes that all items load on the same factor, assuming tau equivalence; that is, it means that the factor loading of all items is equal, which is often not a reasonable assumption. Therefore, McDonald's omega internal consistency assessment method [30] was carried out, which considers that the factor loadings may differ. In the case of NPVS-3, the factor loadings are very similar, suggesting equivalence. The Table 4 below shows factor statistics, including two internal consistency measures showing this similarity between the methods.

**Table 4:** Cronbach’s alpha comparative McDonalds omega internal consistency assessment method

Factor	N	Items	Mean	SD	Min	Max	Alpha	α Lower CI	α Upper CI	Omega	ω Lower CI	ω Upper CI
Caring	**209**	**10**	**40.71**	**5.28**	**16**	**50**	**0.852**	**0.822**	**0.882**	**0.853**	**0.821**	**0.881**
Activism	**209**	**10**	**38.47**	**5.22**	**19**	**50**	**0.832**	**0.798**	**0.866**	**0.833**	**0.798**	**0.865**
Professionalism	**209**	**8**	**30.55**	**3.32**	**17**	**39**	**0.676**	**0.609**	**0.743**	**0.677**	**0.606**	**0.739**

Below is a visualization of the adapted model with the estimated values for factor loadings and correlations between the domains.

**Fig. 1:**
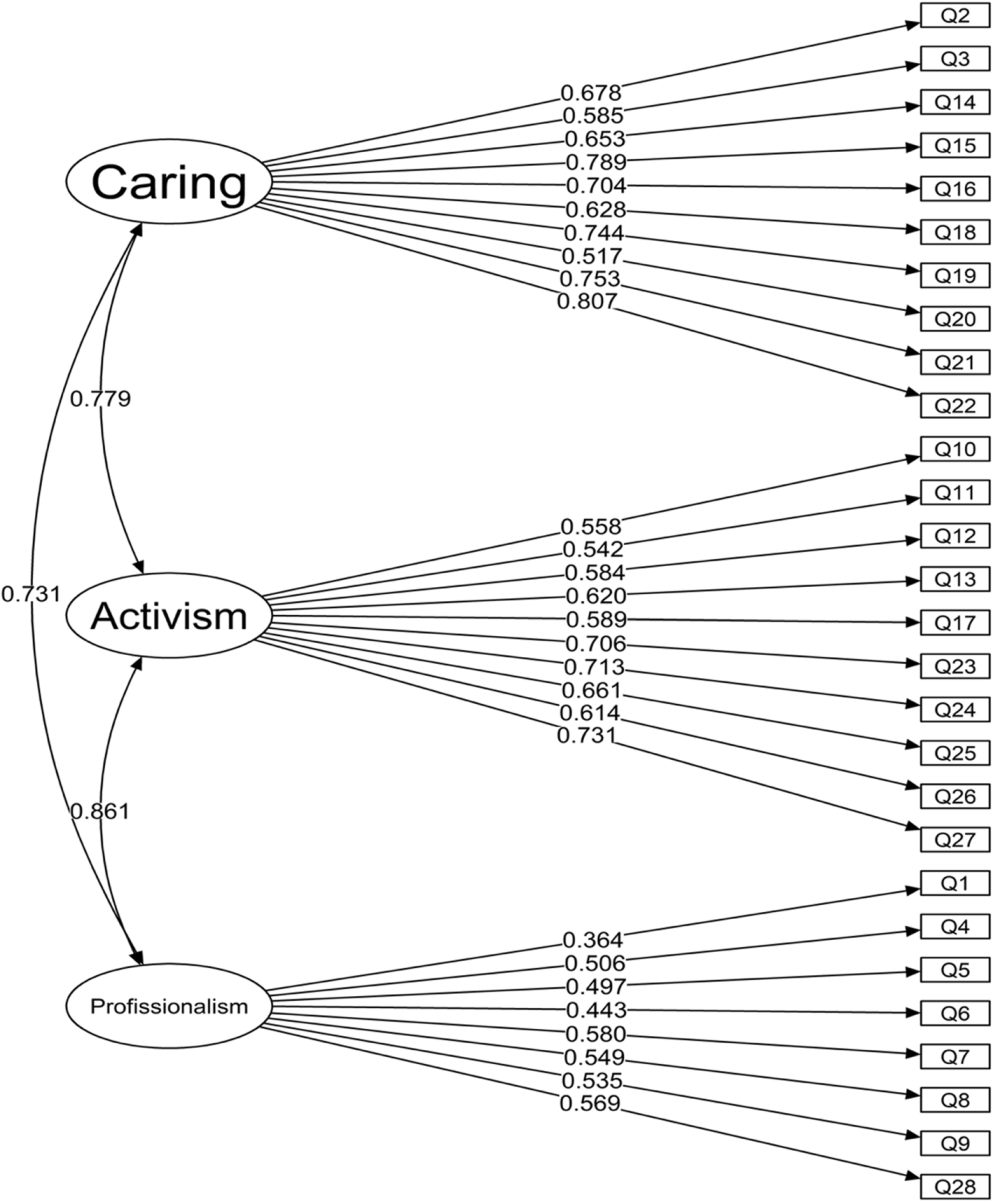
Adjusted model: estimated values for factor loadings and domain correlations

Below is a visualization of the adapted model with the estimated values for factor loadings and correlations between the domains (Fig. 1).

## Discussion

To analyze the professional values of nurses, focusing on their influence and development in the daily practice of the profession, given the constant changes in social and clinical scenarios, and for its use in the Austrian context, this study aims to translate and cross-culturally adapt the NPVS-3; conduct a psychometric evaluation of the NPVS-3, derived from the Code of Ethics for Nurses, which was based on the 2015 Code of Ethics for Nurses [13]. This Code reflects global nursing care concerns regarding patient safety, commitment to the individual, family, and society, personal health, and universal principles and concepts, such as respect for life, dignity, and human rights, without discrimination. This resulted in the three factors (Caring, Activism, and Professionalism). Confirmatory Factor Analysis supported the structure of these three factors, which were developed from the conceptual model. The fit indices approached an adequate level. In the NPVS-3, Caring, the first factor, accounted for the most value variation (41.6%). Caring is central to nursing practice and inherent in the first three provisions of the Brazilian nursing code of ethics [12]. In Factor 1, the principles of fidelity, respect for the individual, responsibility, and responsibility for practice are also found.

Activism, the second factor, accounted for 6.7% of the variance. The last three provisions of the code address responsibilities, reflecting the activist role of the nursing professional—these provisions center on the social nature of the profession and its commitment to society. Nurses can address health challenges and issues. We know that health challenges are broader, more complex, and often long-term, requiring strategic and often multifactorial approaches. Health issues are specific medical problems that typically require immediate attention and treatment. The world faces different health challenges, for example, addressing rising rates of childhood obesity in a community by promoting healthier eating habits and physical activity or managing chronic illnesses like diabetes through lifestyle changes and medical support. There are health problems that people around the world share, for example, dealing with a broken leg, treating a case of pneumonia, or managing asthma symptoms. Protecting and promoting people's health should be a nursing concern. This includes the right to health, civil rights, and human rights. In the Activism factor, there’s also an emphasis on fundamental freedoms, a global awareness of the human condition, including concerns about environmental and social justice, health promotion, as well as the nurse’s role in policy formulation, professional efforts in advancing global health, reducing health disparities, participating in nursing associations, and contributing to research and academic investigation [10, 13].

The Professionalism factor accounted for 4.3% of the variance. This factor addresses duty and loyalty to the profession, including the nurse’s responsibility for nursing practice, leadership in health promotion, and the duty to provide ethical and quality care in a safe environment. Professional duty in nursing refers to the ethical and legal responsibilities nurses must fulfill in their practice. These duties are outlined in international and national codes of ethics, which are fundamental guidelines for nurses' daily conduct and decision-making [12, 13]. Loyalty to the nursing profession encompasses a deep commitment to the values, ethics, and responsibilities inherent to nursing. It manifests itself in several ways, reflecting dedication to patient care: in patient advocacy, nurses need to prioritize the well-being and rights of their patients, advocating for their needs and ensuring they receive the best possible care; compassion and empathy, demonstrating genuine concern and understanding for patients' conditions and experiences, promoting a supportive and healing environment; continuous professional development: lifelong learning, involvement in ongoing education and training to stay up to date with medical advances, new technologies and best practices in nursing; certification and specialization, seeking additional certifications and specializations to improve skills and provide higher levels of care; and adherence to ethical standards: professional ethics, defending the principles outlined in international and national nursing codes of ethics, such as those of the American Nurses Association (ANA), Austria Code, International Council of Nurses (ICN); integrity and honesty, maintaining transparency and honesty in all professional interactions, ensuring the trust and respect of patients and colleagues; defense of the profession: promotion of the profession, actively promoting the value and importance of nursing in the healthcare system and the community at large; participation in professional organizations, engaging with professional nursing organizations to contribute to the advancement of the field and to stay informed about policy changes and innovations [2, 7, 11, 31–33].

The Cronbach Alpha obtained is similar to the original Weis & Schank model [2], which pointed to Factor 1: Caring (0.852), Factor 2: Activism (0.832), and Factor 3: Professionalism (0.676) and the total 0.961.

Other countries that conducted cross-cultural adaptation also obtained similar values, such as in the Arabic version, where the instrument’s Cronbach’s alpha was 0.967 (Factor 1: 0.960, Factor 2: 0.964 and Factor 3: 0.886) [18]; in the Indonesian version, an Cronbach’s alpha of 0.97 was reported (Factor 1: 0.94, Factor 2: 0.95, and Factor 3: 0.89) [19]; in the Turkish version, the overall Cronbach’s alpha was 0.92 (Factor 1: 0.85, Factor 2: 0.73 and Factor 3: 0.86) [20]; in the Chinese version, the Cronbach’s alpha was 0.90 (Factor 1: 0.90, Factor 2: 0.81 and Factor 3: 0.88) [21]; in the Italian version, the Cronbach’s alpha was 0.915 (Factors 1, 2, and 3 ranged between 0.909 and 0.922) [16].

### Limitations

It is also important to acknowledge that this study was conducted with a convenience sample and with a relatively small number of participants. This limits the ability to generalize findings.

## Conclusions

This study concluded that the Austrian version of the NPVS-3 is a valid and reliable instrument for measuring the values of nursing students and professionals in the Austrian context. The average score on the NPVS-3 was 108.4, emphasizing the dimension of Caring, with an average score of 39.98, followed by Activism at 37.92 and Professionalism at 30.49. The construct validity, internal consistency criteria, and close alignment with validations in different cultures, especially the original version of the scale, all contribute to the instrument’s validity.

The researchers’ familiarity with the constructs assessed by the instrument facilitated clarification and assistance with questions and decisions about using certain expressions, ultimately leading to the finalization of the instrument’s version for Austria.

The measurement of professional values among nursing students and nurses provides a foundation for developing and adopting interventions to enhance professional practice. It allows for comparing differences and similarities in values between those on the path to a future in nursing and those already involved in healthcare.

### Supplementary Information

Supplementary Material 1.

Supplementary Material 2.

## Data Availability

The datasets used and/or analyzed during the current study are available from the corresponding author on reasonable request.
