# Supplementary material for: Nursing Professional Values Scale (NPVS-3) in an Austrian context: validation of a scale and reliability assessment
Source: BMC Nurs. 2024 Jul 29;23:510. doi: 10.1186/s12912-024-02175-6 (PMC11288009; doi:10.1186/s12912-024-02175-6)
Supplement: Supplementary file 1 — Supplementary Material 1. [file 12912_2024_2175_MOESM1_ESM.pdf]

Nurses Professional Values Scale-Three (NPVS-3) ©

Indicate the importance of the following value statements relative to nursing practice.
Please fill in the circle next to the degree of importance.

(A = not important to E = most important) for each statement.

	Not important	Somewhat important	Important	Very Important	Most important
	A	B	C	D	E
1. Engage in on-going self-evaluation.	O A	O B	O C	O D	O E
2. Respect the inherent dignity, values, and human rights of all individuals.	O A	O B	O C	O D	O E
3. Protect health and safety of the patient/public.	O A	O B	O C	O D	O E
4. Assume responsibility for personal well-being.	O A	O B	O C	O D	O E
5. Participate in peer review.	O A	O B	O C	O D	O E
6. Establish standards as a guide for practice.	O A	O B	O C	O D	O E
7. Promote and maintain standards where planned learning activities for students take place.	O A	O B	O C	O D	O E
8. Initiate actions to improve environments of practice.	O A	O B	O C	O D	O E
9. Seek additional education to update knowledge and skills to maintain competency.	O A	O B	O C	O D	O E
10. Advance the profession through active involvement in health-related activities.	O A	O B	O C	O D	O E
11. Recognize the role of professional nursing associations in shaping health policy.	O A	O B	O C	O D	O E
12. Establish collaborative partnerships to reduce healthcare disparities.	O A	O B	O C	O D	O E
13. Assume responsibility for meeting health needs of diverse populations.	O A	O B	O C	O D	O E
14. Accept responsibility and accountability for own practice.	O A	O B	O C	O D	O E
15. Protect moral and legal rights of patients.	O A	O B	O C	O D	O E
16. Act as a patient advocate.	O A	O B	O C	O D	O E

Nurses Professional Values Scale-Three (NPVS-3) ©

	Not important	Somewhat important	Important	Very Important	Most important
	A	B	C	D	E
17. Participate in nursing research and/or implement research findings appropriate to practice.	O A	O B	O C	O D	O E
18. Provide care without bias or prejudice to patients and populations.	O A	O B	O C	O D	O E
19. Safeguard patient's right to confidentiality and privacy.	O A	O B	O C	O D	O E
20. Confront practitioners with questionable or inappropriate practice.	O A	O B	O C	O D	O E
21. Protect rights of participants in research.	O A	O B	O C	O D	O E
22. Practice guided by principles of fidelity and respect for person.	O A	O B	O C	O D	O E
23. Actively promote health of populations.	O A	O B	O C	O D	O E
24. Participate in professional efforts and collegial interactions to ensure quality care and professional satisfaction.	O A	O B	O C	O D	O E
25. Promote mutual peer support and collegial interactions to ensure quality care and professional satisfaction.	O A	O B	O C	O D	O E
26. Take action to influence legislators and other policy makers to improve health care.	O A	O B	O C	O D	O E
27. Engage in consultation/collaboration to provide optimal care.	O A	O B	O C	O D	O E
28. Recognize professional boundaries.	O A	O B	O C	O D	O E

Demographics: Fill in the circle next to the appropriate descriptor

29. O Undergraduate Student O Graduate Student O Practicing nurse

30. O Female O Male

31. O African American O Asian/Pacific Islander O White O Hispanic O Native American

© Copyright 2017, Darlene Weis & Mary Schank
