# Supplementary material for: Nursing Professional Values Scale (NPVS-3) in an Austrian context: validation of a scale and reliability assessment
Source: BMC Nurs. 2024 Jul 29;23:510. doi: 10.1186/s12912-024-02175-6 (PMC11288009; doi:10.1186/s12912-024-02175-6)
Supplement: Supplementary file 2 — Supplementary Material 2. [file 12912_2024_2175_MOESM2_ESM.pdf]

Bewertungsskala für berufliche Werte in der Pflege - 3 (NPVS-3)©

Geben Sie die Wichtigkeit der folgenden Wertaussagen in Bezug auf Ihre Pflegepraxis an.
Markieren Sie für jede Aussage den Kreis, der den Grad der Wichtigkeit angibt von (A) unwichtig bis (E) am wichtigsten:

		unwichtig	einigermaßen wichtig	wichtig	sehr wichtig	am wichtigsten
		A	B	C	D	E
1.	Durchführung einer kontinuierlichen Selbstbeurteilung	○	○	○	○	○
2.	Respekt der angeborenen Würde, der Werte und der Menschenrechte aller Personen	○	○	○	○	○
3.	Schutz der Gesundheit und Sicherheit des Patienten/der Öffentlichkeit	○	○	○	○	○
4.	Übernahme von Verantwortung für Ihr persönliches Wohlbefinden	○	○	○	○	○
5.	Teilnahme an Peer Reviews	○	○	○	○	○
6.	Festlegung von Standards als Leitfaden für die Praxis	○	○	○	○	○
7.	Förderung und Aufrechterhaltung von Standards, wo geplante Lernaktivitäten für Studierende stattfinden	○	○	○	○	○
8.	Initiierung von Maßnahmen zur Verbesserung der Praxisumgebung	○	○	○	○	○
9.	Suche nach zusätzlichen Schulungen, um Ihr Wissen und Ihre Fähigkeiten zu aktualisieren und Ihre Kompetenz zu erhalten	○	○	○	○	○
10.	Förderung des Berufsstandes durch aktive Beteiligung an gesundheitsbezogenen Aktivitäten	○	○	○	○	○
11.	Erkennen der Rolle von Berufsverbänden der Krankenpflege bei der Gestaltung der Gesundheitspolitik.	○	○	○	○	○
12.	Aufbau von Kooperationspartnerschaften zum Abbau von Ungleichheiten im Gesundheitswesen	○	○	○	○	○
13.	Übernahme von Verantwortung für die Erfüllung der Gesundheitsbedürfnisse verschiedener Bevölkerungsgruppen	○	○	○	○	○
14.	Übernahme von Verantwortung und Rechenschaftspflicht für die eigene Praxis	○	○	○	○	○
15.	Schutz der moralischen und gesetzlichen Rechte der Patienten	○	○	○	○	○
16.	Handeln als Anwalt des Patienten	○	○	○	○	○
17.	Teilnahme an der Pflegeforschung und/oder praxisgerechten Umsetzung von Forschungsergebnissen	○	○	○	○	○
18.	Versorgung von Patienten und Bevölkerungsgruppen ohne Voreingenommenheit oder Vorurteile	○	○	○	○	○
19.	Schutz des Rechts der Patienten auf Vertraulichkeit und Privatsphäre	○	○	○	○	○
20.	Konfrontation von Praktikern mit fragwürdigen oder unangemessenen Praktiken	○	○	○	○	○
21.	Schutz der Rechte von Forschungsteilnehmern	○	○	○	○	○
22.	Praktiken, die von den Prinzipien der Treue und des Respekts vor der Person geleitet werden	○	○	○	○	○
23.	Aktive Förderung der Gesundheit der Bevölkerung	○	○	○	○	○
24.	Beteiligung an professioneller Arbeit und kollegialem Umgang, um die Qualität der Pflege und der beruflichen Zufriedenheit sicherzustellen	○	○	○	○	○
25.	Förderung der gegenseitigen Unterstützung zwischen Kollegen und im kollegialen Umgang, um die Qualität der Pflege und der beruflichen Zufriedenheit sicherzustellen	○	○	○	○	○
26.	Aktive Ergreifung von Maßnahmen, um Gesetzgeber und andere politische Entscheidungsträger zur Verbesserung der Gesundheitsversorgung zu bewegen	○	○	○	○	○
27.	Engagement bei der Beratung/Zusammenarbeit zur Gewährleistung einer optimalen Versorgung	○	○	○	○	○

	unwichtig	einigermaßen wichtig	wichtig	sehr wichtig	am wichtigsten
	A	B	C	D	E
28. Erkennen von beruflichen Grenzen	○	○	○	○	○

**Demografische Daten: Markieren Sie den Kreis neben der entsprechenden Option**					
29.	○ Absolvent*in		○ Postgraduiert	○ Student*in der Krankenpflege im ___. Semester	
30.	○ weiblich		○ männlich	○ divers	
31.	○ weiß*	○ schwarz	○ gemischt	○ gelb	○ indigen

*) Angepasst nach der Nomenklatur des IBGE für die demografische Volkszählung: https://sidra.ibge.gov.br/tabela/2094

© Copyright 2017, Darlene Weis & Mary Schank
